# Complete Dentures Manufactured Using CAD/CAM Technology and a Modified Individual Tray Recording Method

**DOI:** 10.1155/crid/6224091

**Published:** 2025-08-07

**Authors:** Paweena Kongkon, Awutsadaporn Katheng, Piangkwan Saiprasert, Pokpong Amornvit

**Affiliations:** ^1^Department of Restorative Dentistry, Faculty of Dentistry, Naresuan University, Phitsanulok, Thailand; ^2^PPFACEDESIGN, The S Clinic, Bangkok, Thailand

**Keywords:** complete denture, digital denture, modified individual tray

## Abstract

This study introduces the Tako tray, a modified individual tray designed for the closed-mouth impression technique, and demonstrates its integration with Ivotion CAD/CAM technology to enhance complete denture fabrication by combining conventional and digital workflows. A clinical case was conducted using the Tako tray to facilitate accurate impression-taking and jaw relation recording. Primary impressions were taken, followed by the fabrication of the Tako tray. The closed-mouth definitive impression was recorded in centric relation, simultaneously capturing functional and jaw relation records. The collected data were digitized and processed using the Ivotion CAD/CAM system for virtual denture design and milling. This approach streamlined the fabrication process and minimized patient visits. The integration of the Tako tray with the Ivotion workflow reduced clinical appointments to three, while improving occlusal rim retention and ensuring accurate jaw relation recording. The final dentures demonstrated precise fit, functional efficiency, and satisfactory esthetics, with the patient reporting high satisfaction. The combination of the Tako tray and digital technology optimized denture fabrication by increasing efficiency, reducing patient chair time, and achieving predictable, high-quality outcomes. Clinically, the Tako tray simplifies the closed-mouth impression technique, making digital workflows more accessible for complete denture cases while enhancing patient comfort and improving prosthetic results.

## 1. Introduction

Traditionally, the fabrication of complete dentures requires four to five visits before the final prosthesis is delivered to the patient. The process typically begins with the first visit, where a primary impression is taken using alginate. During the second visit, border molding is performed using an individual tray, followed by the final impression using elastomeric material. The third visit involves the occlusion rim try-in and the recording of the jaw relation through bite registration. A denture try-in is optionally performed during the fourth visit to ensure proper fit and esthetics. Finally, the completed denture is delivered to the patient during the last visit. In contrast, the digital approach to complete denture fabrication streamlines this process, reducing the number of visits to just three: the primary impression using an intraoral scanner, occlusion rim try-in, final impression and bite registration, and denture delivery. During the second visit, the individual tray serves a dual purpose, functioning as both the tray for impression materials and the baseplate for the occlusion rim [1–3]. A comparison of the clinical workflows for conventional and digital complete denture fabrication is shown in Table 1. To enhance the retention of the occlusion rim on the tray, modifications are made to enhance its design. The Tako tray is a modified individual tray that adds several poles to improve the retention of the occlusion rim.

## 2. Case Presentation

This case report presents a 65-year-old male who sought dental care due to difficulties in chewing food. The patient has a significant medical history, including coronary artery disease. The intraoral examination revealed that the upper and lower edentulous areas were within normal limits, exhibiting a U-shaped form with firm consistency. No signs of inflammation were observed in the surrounding soft tissues. The patient was informed that a complete denture would be fabricated using CAD/CAM technology. The clinical and laboratory steps involved in the process are presented below.

## 3. Procedure

**Figure 1:**
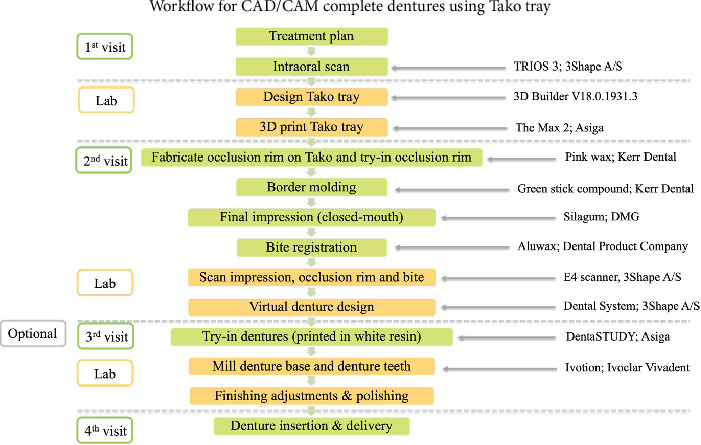
Clinical workflows for digital complete denture fabrication.

**Figure 2:**
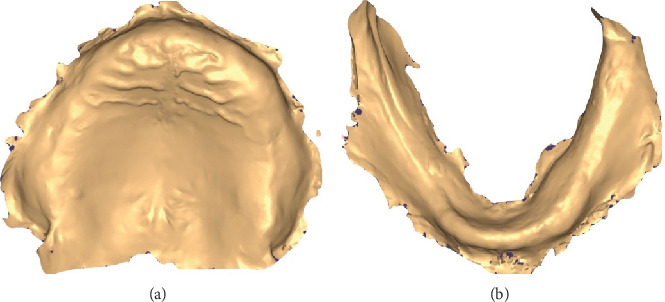
Digital file of the edentulous jaws. (a) Upper jaw. (b) Lower jaw.

**Figure 3:**
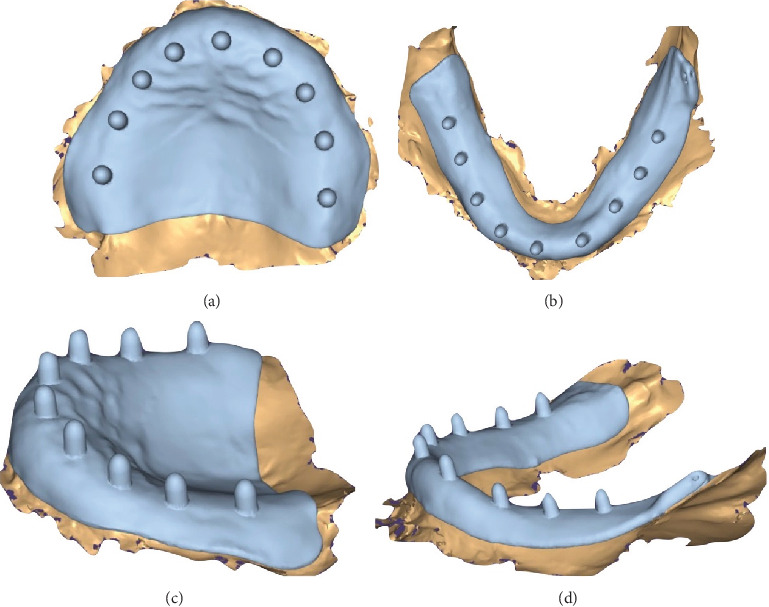
Digital design of the Tako tray with cylindrical poles. (a) Maxillary occlusal view. (b) Mandibular occlusal view. (c) Maxillary lateral view. (d) Mandibular lateral view.

**Figure 4:**
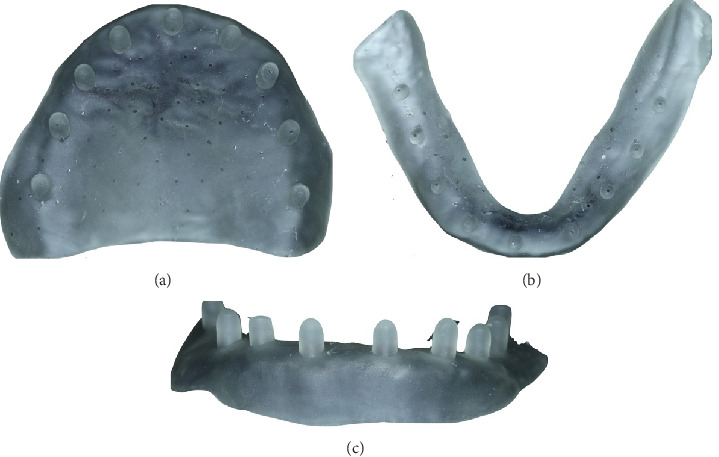
Clear resin Tako tray with poles. (a) Maxillary occlusal view. (b) Mandibular occlusal view. (c) Maxillary frontal view.

**Figure 5:**
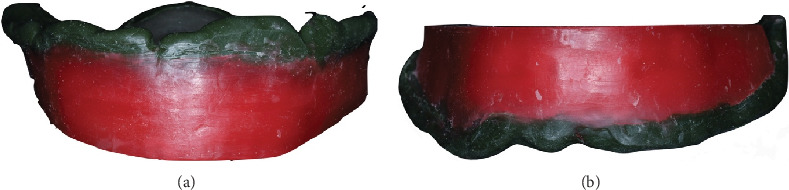
Baseplate-occlusal rims with border molding using green stick compound. (a) Upper arch. (b) Lower arch.

**Figure 6:**
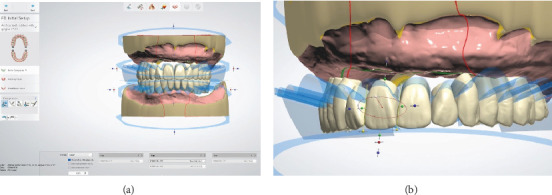
Virtual teeth set up. (a) Teeth with individual corrections in size, shape, and position. (b) The scallop line showed the teeth-base junction.

**Figure 7:**
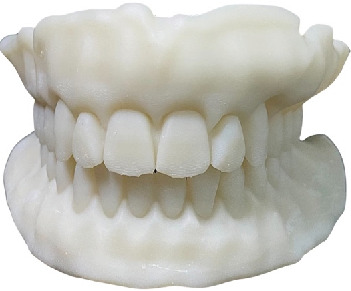
Printed try-in dentures.

**Figure 8:**
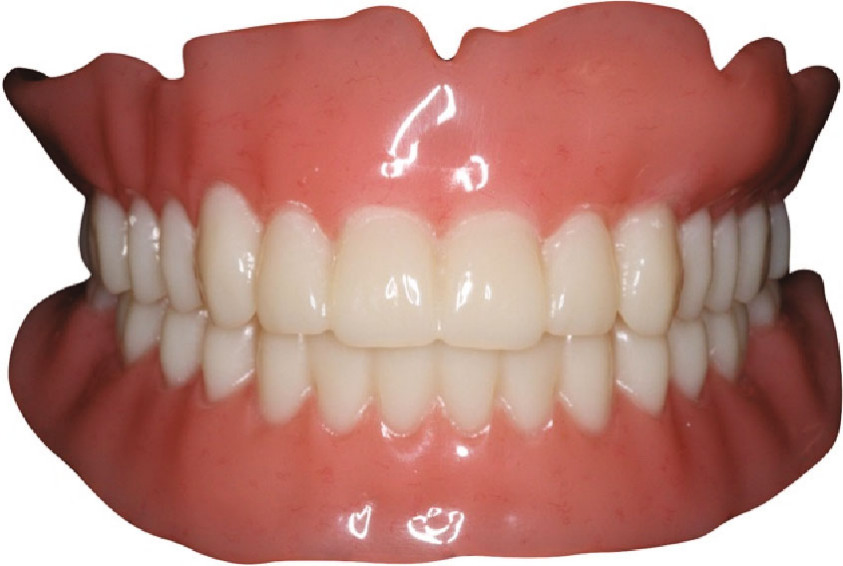
The digitally manufactured CD after the finishing and polishing procedures.

**Figure 9:**
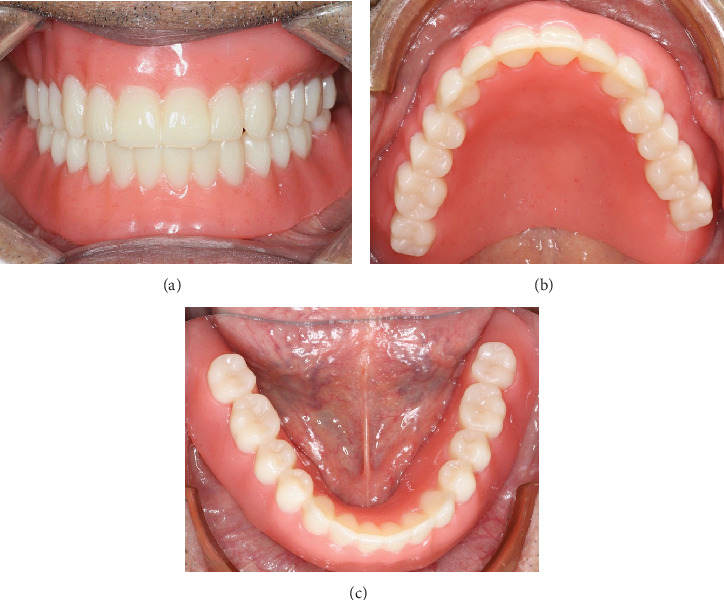
Insertion and delivery of complete denture. (a) Frontal view. (b) Maxillary occlusal view. (c) Mandibular occlusal view.

The steps for fabricating complete dentures using CAD/CAM technology are shown in Figure 1 and include the following:

1. In the first visit, the digital impression step in CAD/CAM for complete dentures involves capturing the patient's oral anatomy using an intraoral scanner (TRIOS 3; 3Shape A/S) (Figure 2). For the upper arch, begin scanning at one tuberosity and proceed to the other, followed by a palatal scan along the midline with a side-to-side movement. Conclude the scan by capturing the buccal aspects, starting from the tuberosity area and moving toward the midline. For the lower arch, start by scanning the lingual aspects from the retromolar area toward the midline, then return along the buccal aspect. Repeat the process on the other side.
2. Identically sized and shaped, the Tako tray was designed using CAD software (3D Builder Version 18.0.1931.3, Microsoft Corporation). The Tako tray was designed following the same principles as conventional individual trays. The border extension of the Tako tray was 2 mm above the vestibule. On the occlusal surface of the tray, cylindrical poles with a diameter of 3 mm and a length of 5 mm were added, featuring rounded edges (Figure 3). The tissue surface of the Tako tray can be modified according to the desired impression technique.
3. The Tako tray was then printed using a DLP 3D printer (the MAX 2; Asiga) with a surgical guide resin clear color (Asiga DentaGUIDE; Asiga) and a layer thickness of 50 *μ*m. The light source used was a 405-nm UV LED with a laser power of 13.114 mW/cm^2^ (Figure 4).
4. In the second visit, dental wax (pink wax; Kerr Dental) was used to fabricate the occlusion rims on the Tako tray first. It was important to ensure that the rims represented the anticipated position of the teeth. The rims were then adjusted to the correct height (occlusal vertical dimension) and width according to the patient's facial proportions. Esthetics were evaluated, and the correct vertical dimension and centric relation of the occlusion rim were verified (Figure 5). After that, border molding was performed using an impression compound (green stick compound; Kerr Dental) to capture the functional depth and width of the denture periphery. This ensured a proper seal and fit of the final denture.
5. Confirm that the bite registration was taken using Aluwax (Aluwax Dental Product Company) and that the final impression was made using a light-bodied silicone impression material (Silagum; DMG) to capture fine details by closed-mouth technique.
6. Ensure that the impression was clean and free of any debris or excess material. Calibrated the scanner following the manufacturer's instructions. The impression and bite registration were converted into Standard Tessellation Language (STL) file using Lab scanner (E4 scanner, 3Shape A/S). The scanner typically rotated and captured multiple images from different angles to create a 3D model of the impression. The accuracy of this scanning process was crucial for ensuring a precise fit for the final dentures.
7. In the virtual design step of CAD/CAM for complete dentures, the digital data from the scanning process was used to create a 3D model of the denture. Using CAD software (Dental System; 3Shape A/S), the dental technician or dentist designed the denture base and arranged the artificial teeth virtually (Figure 6). This approach allowed for precise adjustments to ensure proper fit, occlusion, and esthetics.
8. According to Ivotion disk (Ivotion; Ivoclar Vivadent), CAD software was needed to design the complete denture according to the impression and occlusal rim. The scallop blue line (shell geometry) indicated the junction between the teeth and the base, which may be limited by the Ivotion disk.
9. In the third visit (optional), the try-in dentures (Figure 7) were printed with white resin (DentaSTUDY; Asiga) following the manufacturer's instructions. This step allowed the clinician to evaluate the denture and check for any possible errors, such as midline position, occlusion, and phonetics. If any errors were identified, the try-in dentures were used as a reference for redesign.
10. In the milling step of CAD/CAM for complete dentures, the denture base was created by machining the Ivotion disk. This process used a computer-controlled milling machine that precisely carved the denture base according to the digital design. The material used was typically a high-quality resin that mimicked the appearance and strength of traditional denture bases. This method ensures a highly accurate fit, reducing the need for manual adjustments and enhancing the overall quality and durability of the final denture.
11. In the finishing step of CAD/CAM complete denture fabrication (Figure 8), the dentures undergo final adjustments and polishing to ensure optimal fit, function, and esthetics. This includes checking for any rough edges, making necessary modifications to enhance comfort, and refining the occlusion to ensure proper bite alignment. The dentures were then polished to achieve a smooth and glossy surface, enhancing their appearance and making them more comfortable to wear. This final step ensured that the dentures were ready for insertion and long-term use by the patient.
12. In the fourth visit, in the insertion and delivery step, the dentures were fitted into the patient's mouth (Figure 9). The dentist checked for comfort, fit, and function, ensuring that the dentures aligned properly with the patient's bite and that they did not cause any discomfort. Follow-up visits were necessary for further adjustments.

## 4. Outcomes

The application of the Tako tray in combination with Ivotion technology effectively streamlined the digital complete denture workflow, achieving high accuracy while reducing the number of patient visits. The closed-mouth impression technique using the Tako tray provided a functional and detailed impression, leading to an improved denture fit and stability. The addition of cylindrical poles on the occlusal surface of the tray enhanced the retention of occlusion rims, ensuring a more precise maxillomandibular relationship during bite registration. The final prosthesis demonstrated excellent adaptation to the edentulous ridge, reducing the need for significant postinsertion adjustments. The integration of CAD/CAM technology resulted in a well-defined denture base and occlusion, minimizing errors common in conventional fabrication methods. The milling process using the Ivotion monolithic disk contributed to a uniform and durable prosthesis, enhancing longevity and structural integrity. The patient reported a significant improvement in chewing ability and overall satisfaction with the comfort and esthetics of the denture. The reduction in chair time and the simplified clinical workflow made the treatment more efficient for both the patient and the dental team. However, challenges such as the height limitation of the Ivotion disk and the need for careful selection of materials for 3D printing were identified.

Despite the higher initial cost of digital fabrication, the long-term benefits of precision, reproducibility, and efficiency suggest a promising future for integrating hybrid techniques in complete denture fabrication. The results highlight the potential for further refinement and adaptation of digital workflows to enhance clinical outcomes and patient experience.

## 5. Discussion

CAD/CAM technology in complete denture fabrication represents a significant advancement in prosthodontics, providing an efficient and streamlined alternative to conventional methods. The process involves digital scanning of impressions and bite registrations, followed by virtual modeling of the dentures. This approach not only improves the precision of denture base milling but also reduces chair time significantly, enhancing the overall patient experience [4–6]. One key benefit is the ability to store digital data for future use, making replacements or adjustments more straightforward. However, it lacks a trial phase for esthetics and function, which can be a drawback [5, 7]. There are many advantages of digital workflow in complete denture fabrication. The first advantage is fewer visits were taken until the denture can be delivered. Compared to conventional complete dentures, digital complete dentures can be delivered in just three visits [1, 8, 9]. Furthermore, digital dentures demonstrated better precision in fabrication than conventional dentures, as they were either milled or 3D printed, resulting in significantly fewer errors than conventional flasking methods [10–12]. Additionally, there are many ways to combine traditional treatment methods with digital approaches. The choice of a CAD/CAM denture fabrication system and how to integrate conventional and digital workflows depends on the dentist's experience in prosthodontics and the specific needs of each patient [13, 14]. Ivoclar's digital denture solution is highly regarded for effectively combining conventional denture principles with modern digital workflows, ensuring both efficiency and high-quality results for patients and dental professionals alike [15, 16]. This approach reduces manual steps, enhances precision, and typically requires only minimal adjustments after fabrication, thereby improving overall efficiency for clinicians and laboratories [17–20].

In this case report, we integrated conventional and digital techniques by designing a Tako tray in combination with Ivotion denture. This approach reduced costs associated with purchasing specialized tools directly from Ivoclar Vivadent company. With the use of Ivotion denture, Ivotion utilizes a monolithic disk that combines both tooth and denture base materials, allowing the fabrication of high-quality, customized complete dentures through a single uninterrupted milling process. This reduced the potential for errors associated with traditional multistep methods. Moreover, the two-colored monolithic disk of Ivotion, used for both teeth and denture base, is copolymerized during the industrial fabrication process. This forms a direct chemical bond, eliminating the need for additional bonding materials and concerns about bond failure between the resin teeth and denture base [21–23]. However, in the case of an Ivotion denture, the height of the denture is limited by the manufacturer, which is currently 38 mm at maximum. Ivotion's technology includes features like shell geometry, which is also another limitation that can affect the overall esthetic of the denture because the junction between the teeth and the base may not be fully customized for every individual tooth.

The Tako tray helps in improving the retention between the occlusion rim and the individual tray by adding multiple poles on the occlusal surface of the tray. The poles are approximately 3 mm wide and 5 mm high, and the border of the Tako tray should be 2 mm away from the vestibule for border molding. However, various materials and printers are commercially available, with differences in precision and trueness [24]. Thus, selecting the appropriate materials and printers for the Tako tray can minimize unnecessary errors. The limitation of this technique is the higher cost of materials and equipment compared to conventional complete dentures. Additionally, there is a lack of studies on appropriate repair and reline methods for digital dentures, which could impact long-term patient outcomes.

## 6. Conclusions

The use of the Tako tray combined with Ivotion CAD/CAM technology offers an effective approach to streamline complete denture fabrication. This hybrid technique enhances clinical efficiency, reduces patient visits, and delivers predictable and satisfactory outcomes, demonstrating the potential of digital integration in conventional complete denture workflows.

## Figures and Tables

**Table 1:** A comparison of the clinical workflow involved in conventional and digital methods for complete denture fabrication.

**Visit**	**Conventional method**	**Digital method (CAD/CAM)**
First	Primary impression with alginate	Primary impression using intraoral scanner
Second	Border molding Final impression	Occlusion rim try-in Border molding Final impression Bite registration
Third	Occlusion rim try-in Bite registration	Teeth try-in (optional)
Fourth	Teeth try-in (optional)	Insertion and delivery
Fifth	Insertion and delivery	—

## Data Availability

The authors have nothing to report.

## References

[1] Kouveliotis G., Tasopoulos T., Karoussis I., Silva N. R., Zoidis P. (2022). Complete Denture Digital Workflow: Combining Basic Principles With a CAD-CAM Approach. *Journal of Prosthetic Dentistry*.

[2] Janeva N., Kovacevska G., Janev E. (2017). Complete Dentures Fabricated With CAD/CAM Technology and a Traditional Clinical Recording Method. *Journal of Medical Sciences*.

[3] Casucci A., Cagidiaco E. F., Verniani G., Ferrari M., Borracchini A. (2025). Digital vs. Conventional Removable Complete Dentures: A Retrospective Study on Clinical Effectiveness and Cost-Efficiency in Edentulous Patients: Clinical Effectiveness and Cost-Efficiency Analysis of Digital Dentures. *Journal of Dentistry*.

[4] Mubaraki M. Q., Moaleem M. M. A., Alzahrani A. H. (2022). Assessment of Conventionally and Digitally Fabricated Complete Dentures: A Comprehensive Review. *Materials*.

[5] Janeva N. M., Kovacevska G., Elencevski S., Panchevska S., Mijoska A., Lazarevska B. (2018). Advantages of CAD/CAM Versus Conventional Complete Dentures - A Review. *Journal of Medical Sciences*.

[6] Kanazawa M., Iwaki M., Arakida T., Minakuchi S. (2018). Digital Impression and Jaw Relation Record for the Fabrication of CAD/CAM Custom Tray. *Journal of Prosthodontic Research*.

[7] Peroz S., Peroz I., Beuer F., Sterzenbach G., von Stein-Lausnitz M. (2022). Digital Versus Conventional Complete Dentures: A Randomized, Controlled, Blinded Study. *Journal of Prosthetic Dentistry*.

[8] Chappuis Chocano A. P., Venante H. S., Bringel da Costa R. M., Pordeus M. D., Santiago Junior J. F., Porto V. C. (2023). Evaluation of the Clinical Performance of Dentures Manufactured by Computer-Aided Technology and Conventional Techniques: A Systematic Review. *Journal of Prosthetic Dentistry*.

[9] Thu K. M., Molinero-Mourelle P., Yeung A. W. K., Abou-Ayash S., Lam W. Y. H. (2024). Which Clinical and Laboratory Procedures Should Be Used to Fabricate Digital Complete Dentures? A Systematic Review. *Journal of Prosthetic Dentistry*.

[10] Wang C., Shi Y. F., Xie P. J., Wu J. H. (2021). Accuracy of Digital Complete Dentures: A Systematic Review of In Vitro Studies. *Journal of Prosthetic Dentistry*.

[11] Zandinejad A., Floriani F., Lin W. S., Naimi-Akbar A. (2024). Clinical Outcomes of Milled, 3D-Printed, and Conventional Complete Dentures in Edentulous Patients: A Systematic Review and Meta-Analysis. *Journal of Prosthodontics*.

[12] Gad M. A., Abdelhamid A. M., ElSamahy M., Abolgheit S., Hanno K. I. (2024). Effect of Aging on Dimensional Accuracy and Color Stability of CAD-CAM Milled and 3D-Printed Denture Base Resins: A Comparative In-Vitro Study. *BMC Oral Health*.

[13] Schweiger J., Stumbaum J., Edelhoff D., Güth J. F. (2018). Systematics and Concepts for the Digital Production of Complete Dentures: Risks and Opportunities. *International Journal of Computerized Dentistry*.

[14] Steinmassl P.-A., Klaunzer F., Steinmassl O., Dumfahrt H., Grunert I. (2017). Evaluation of Currently Available CAD/CAM Denture Systems. *International Journal of Prosthodontics*.

[15] Villias A., Karkazis H., Yannikakis S., Theocharopoulos A., Sykaras N., Polyzois G. (2021). Current Status of Digital Complete Dentures Technology. *Prosthesis*.

[16] Avelino M. E. L., Costa R. T. F., Vila-Nova T. E. L., Vasconcelos B., Pellizzer E. P., Moraes S. L. D. (2024). Clinical Performance and Patient-Related Outcome Measures of Digitally Fabricated Complete Dentures: A Systematic Review and Meta-Analysis. *Journal of Prosthetic Dentistry*.

[17] Srinivasan M., Kamnoedboon P., McKenna G. (2021). CAD-CAM Removable Complete Dentures: A Systematic Review and Meta-Analysis of Trueness of Fit, Biocompatibility, Mechanical Properties, Surface Characteristics, Color Stability, Time-Cost Analysis, Clinical and Patient-Reported Outcomes. *Journal of Dentistry*.

[18] Maragliano-Muniz P., Kukucka E. D. (2021). Incorporating Digital Dentures Into Clinical Practice: Flexible Workflows and Improved Clinical Outcomes. *Journal of Prosthodontics*.

[19] Liao P., Budsabong O. (2024). A Method of Fabricating a Stackable CAD-CAM Custom Record Tray for Complete Dentures. *Journal of Prosthetic Dentistry*.

[20] Qu F., Du X., Liu W. C. (2019). 3D-Printed Custom Trays With a Gothic Arch for Centric Relation Recording and Definitive Impression Making for Complete Dentures: A Dental Technique. *Journal of Prosthetic Dentistry*.

[21] Tzanakakis E.-G., Pandoleon P., Sarafianou A., Kontonasaki E. (2023). Adhesion of Conventional, 3D-Printed and Milled Artificial Teeth to Resin Substrates for Complete Dentures: A Narrative Review. *Polymers*.

[22] Mohamed A., Takaichi A., Kajima Y., Takahashi H., Wakabayashi N. (2023). Bond Strength of CAD/CAM Denture Teeth to a Denture Base Resin in a Milled Monolithic Unit. *Journal of Prosthodontic Research*.

[23] Kane B., Shah K. C. (2023). In Vitro Analysis of Shear Stress: CAD Milled vs Printed Denture Base Resins With Bonded Denture Tooth. *Journal of Prosthodontics*.

[24] Al-Qarni F. D., Gad M. M. (2022). Printing Accuracy and Flexural Properties of Different 3D-Printed Denture Base Resins. *Materials (Basel)*.

